# LC-MS Based Analysis and Biological Properties of *Pseudocedrela kotschyi* (Schweinf.) Harms Extracts: A Valuable Source of Antioxidant, Antifungal, and Antibacterial Compounds

**DOI:** 10.3390/antiox10101570

**Published:** 2021-10-02

**Authors:** Kouadio Ibrahime Sinan, Stefano Dall’Acqua, Irene Ferrarese, Adriano Mollica, Azzurra Stefanucci, Jasmina Glamočlija, Marina Sokovic, Marija Nenadić, Abdurrahman Aktumsek, Gokhan Zengin

**Affiliations:** 1 Physiology and Biochemistry Research Laboratory, Department of Biology, Science Faculty, Selcuk University, Konya 42130, Turkey; aktumsek@selcuk.edu.tr; 2 Department of Pharmaceutical and Pharmacological Sciences, University of Padova, Via Marzolo 5, 35131 Padova, Italy; irene.ferrarese@unipd.it; 3 Department of Pharmacy, G. d’Annunzio University of Chieti-Pescara, Via dei Vestini 31, 66100 Chieti, Italy; a.mollica@unich.it (A.M.); a.stefanucci@unich.it (A.S.); 4 Laboratory of Mycology, Department of Plant Physiology, Institute for Biological Research “Siniša Stanković”, National Institute of Republic of Serbia, University of Belgrade, 11000 Belgrade, Serbia; jasna@ibiss.bg.ac.rs (J.G.); marina.sokovic@mpn.gov.rs (M.S.); marija.nenadic@bio.bg.ac.rs (M.N.)

**Keywords:** *Pseudocedrela kotschyi*, LC-MS^n^, antioxidants, enzyme inhibition, antimicrobial activity, multi-block discriminant analysis

## Abstract

The impact of two extraction solvents on the phenolic composition, antioxidant, and enzymes inhibitory and antimicrobial activities of two parts (leaves and stem bark) of *P. kotschyi* was studied. Two different LC-DAD-MS^n^ approaches were used to identify and quantify the bioactive compounds in the different extracts. A total of thirty-two compounds were quantified, being the procyanidin the most abundant in stem bark while catechin and flavonoids are most abundant in leaves. Overall, the stem bark extraction using methanol showed higher amounts of total phenolic (131.83 ± 1.81 mg GAE/g) and flavanol (14.14 ± 0.11 mg CE/g) while the leaves extraction using water exhibited stronger levels of total flavonoid (44.95 ± 0.38 mg RE/g) and phenolic acid (63.58 ± 2.00 mg CAE/g). As regards the antioxidant assays, methanol stem bark extracts were characterized by the highest antioxidant activities (DPPH: 1.94 ± 0.01 mmol TE/g, ABTS: 3.31 ± 0.01 mmol TE/g, FRAP: 2.86 ± 0.02 mmol TE/g, CUPRAC: 5.09 ± 0.08 mmol TE/g, phosphomolybdenum: 5.16 ± 0.23 mmol TE/g and metal chelating: 17.12 ± 0.46 mg EDTAE/g). In addition, the methanolic extracts of stem bark had highest impact on acetylcholinesterase (2.54 mg GALAE/g), butyrylcholinesterase (5.48 mg GALAE/g). In contrast, the methanolic extracts of leaves was potent against tyrosinase (77.39 ± 0.21 mg KAE/g) and α-glucosidase (0.97 ± 0.01 mmol ACAE/g), while a higher anti-α–amylase (0.97 ± 0.01 mmol ACAE/g) was observed for water extracts of the same part. All of the tested extracts showed inhibitory effects on elastase, except methanolic leaves extracts. Additionally, the extracts exhibited appreciable antifungal toward *A. ochraceus*, *A. fumigatus*, *P. ochrochloron*, *T. viride,* and *P. funiculosum* and promising antibacterial activity against *M. flavus*, *S. aureus*, *L. monocytogenes*, *E. coli*, *P. aeruginosa*, *E. cloacae*, and *S. typhimurium.* Taken together, the outcomes demonstrated *P. kotschyi* as a novel source of bioactive molecules of interest with an evident therapeutic value.

## 1. Introduction

Herbal based medicines and remedies have a lengthy history of use on all continents for the therapy of several complaints and diseases. Medicinal plants are highly diffused, both for the traditional medicines, as well as for the development of new treatments, and scientific studies assessing their composition and efficacy are needed. The available knowledge on the utilization of herbals preparations in folk medicine is large but still too empiric. The evaluation of the bioactivities of complex mixtures as extracts can be a challenge, and the difference in chemical composition can seriously afflict final effects. Many herbal medicines used in traditional preparation are only poorly studied for their chemical content and for their potential usefulness in therapy. On the other hand, the large amount of traditional knowledge that is present in many countries about the medicinal plant is still orally transmitted and there is a risk of losing information. For these reasons, scientific studies on traditional species are urgently needed to evaluate these botanicals as sources of bioactive compounds, as well as to substantiate their importance in the traditional medicine, finally improved knowledge on species, both from a chemical and pharmacological point of view can help biodiversity protection and increase the value of each country’s botanical diversity. Africa is reputed for the exceptional richness of its flora. Based on rigorous ethnobotanical investigations, together with judicious selection of plants, this large botanical biodiversity can be considered a rich source of new natural bioactive compounds being good candidates for the preparation of new drugs. It is well known the importance of the whole natural product in drug discovery and development [1,2]. In this regard, plant derived natural products still play a crucial role [3,4].

Different classes of secondary metabolites present significant pharmacological activities and, thus, to assess the potential bioactivity of botanical species detailed chemical characterization is needed. Secondary metabolite production is needed for plant protection and relationships with other organisms, such as herbivores, phytofages, insects, furthermore, secondary metabolites can help plants in protection due to abiotic or biotic stresses. As example, the protection role of plant chemical compounds, such as polyphenols, against different disorders has been related in several publications [5,6], and numerous new plant derived drugs increased likewise.

The Meliaceae, one of the most studied families, is rich sources of structurally diversified limonoid [7]. This group of molecules draw great interest due to their pharmaceutical properties [8,9,10]. Amongst approximately 1400 species in this family [11], our attention was focused on the species *Pseudocedrela kotschyi. P. kotschyi* is used in different parts of Africa by traditional healers in treatment of epilepsy, diabetes, diarrhea, rheumatism, aphrodisiac, anemia, and menstruation disorder [12,13]. Some of these properties have been highlighted in several scientific studies. For example, the antibacterial, anticonvulsant, antidiabetic, antifungal, antimalarial, anti-inflammatory, anti-analgesic, antipyretic, antiulcer, antidiarrhoeal, and anticancer activities have been reported [13,14,15,16,17,18,19]. At the same time, the phytochemical screening revealed that roots, stem bark, or leaves contain flavonoids, tannins, saponins, terpenoids, steroids, anthraquinones, and alkaloids [13,20]. In addition, some compounds, such as pseudocedrelin, quercetin, pseudrelones A, B, C, myricetin, stigmasterol, and kotschyins A-C, have been identified in different extracts of the roots, stem bark, or leaves [17,21,22,23,24,25].

As can be noted, the number of publications have been reported on the potential pharmaceutical properties of *P. kotschyi*. Nevertheless, limited studies carrying on both leaves and stem bark have been done. Likewise, no reports are available on the effects of different extraction solvents on the nature and amount of phytochemicals extracted and the pharmaceutical activities. Generally, when plant-derived phytochemicals or pharmaceutical activities are studied, the solvents used for extraction, as well as the parts must be carefully considered. For these reasons, in this work, two extraction solvents (aqueous vs. methanol) and parts (leaves vs. stem bark) were comparatively evaluated according to their antioxidant, enzyme inhibitory, and antimicrobial activities. LC-MS^n^ analysis was also carried out to determine the phytochemicals composition of the extracts. From our point of view, this is the first attempt that deals with an exhaustive analysis of secondary metabolites extracted from stem bark and leaves of *P. kotschyi* collected in Côte d’Ivoire.

## 2. Materials and Methods

### 2.1. Plant Materials

The collecting of the leaves and stem bark of *P. kotschyi* were provided in the village of Prikro (city of Brobo, Côte d’Ivoire), in January 2019. The plant was taxonomically identified by a botanist from the national floristic center (Université Félix Houphouet Boigny, Abidjan, Côte d’Ivoire) and representative specimen was deposited at the herbarium. The two parts were dried at 25 °C temperature for two weeks. Then, they were milled in a laboratory and the obtained thin powder were preserved in room temperature until analysis.

### 2.2. Preparation of Extracts

Maceration: 5 g of leaves or stem bark was extracted with 100 mL of methanol, brought to room temperature and stirred for 24 h. Resulting extracts were filtered and the solvent were evaporated through the use of rotary-evaporator. Infusion: 100 mL boiled water was added to 5 g of each parts. After 20 min the obtained solutions were filtered and lyophilized. All extracts were kept at 4 °C until the further analysis.

### 2.3. Determination of Secondary Metabolites

For each extract, different spectrophotometrical tests, were carried out to quantify the flavonoid, phenolic acid, flavanol, and phenolic compounds. The details of the different methods are available in our previous manuscripts [26,27]. Rutin (RE), caffeic acid (CE), catechin (CAE), or gallic acid (GAE) were used as standards and results were expressed as milligrams RE, CE, CAE, or GAE per grams dry matter, respectively.

### 2.4. LC-MS^n^ Analysis

Samples were analyzed using an LC-DAD-MS^n^ system. Briefly, leaves extracts, and stem bark extracts were weighted and extracted (20 mg/mL methanol) and sonicated for 10 min. Samples were centrifuged at 13,000 rpm for 15 min and liquids were used for LC analysis. The LC-MS^n^ system consisted of an Agilent 1260 quaternary pump coupled to both 1260 Agilent diode array detector (DAD) and a Varian MS 500 Mass spectrometer equipped with Electrospray (ESI) ion. MS data were acquired both in positive and negative ion mode, in the *m/z* range 100 to 2000. Fragmentation pattern of most intense ion species was obtained using the turbo data depending on scanning (TDDS) function of the instrument. MS parameters were as follows: needle voltage, 4.9 kV; shield voltage, 600 V; capillary voltage, 80 V; RF loading, 80%; nebulizing gas pressure, 25 psi (nitrogen); drying gas pressure, 15 psi; drying gas temperature, 300 °C. Identification of compounds was obtained based on comparison with the literature and reference compounds, when available.

The sample solutions were centrifugated for 10 min, then transferred in vial for analysis. An Agilent Eclipse XDB-C18 column (3.0 mm × 150 mm, 3.5 µm) was used as stationary phase.

For mobile phase solvents were water 1% formic acid (A), Acetonitrile (B), and methanol (C). Gradients start with 98% A and 2% B. In 5 min, 90% A, 8% B, and 2% C, then, at 20 min, 70% A, 28% B, and 2% C isocratic up to 25 min. In 5 min, 80% B and 20% C isocratic up to 34 min. The flow rate was 400 µL/min. Injection volume was 10 µL and the temperature was set at 30 °C. Identification of compounds was obtained based on comparison with the literature and reference compounds, when available. Quinic acid, gallic acid, rutin, quercetin 3-O-glucoside, procyanidin B2 were used as external standard and for compound quantification a calibration curve were performed: for rutin at 350 nm and calibration curve was y = 18.03x + 21.1 (R2 = 0.9991), for gallic acid at 280 nm and calibration curve was y = 5.03 x + 0.2 (R2 = 0.9989), for procyanidin B2 at 280 nm y = 4.1x + 0.34 (R2 = 0.9992).

Accurate *m*/*z* values were obtained using a Waters Acquity UPLC system coupled to a Waters Xevo G2 Q-TOF MS detector, operating in ESI (-) mode. For chromatographic separation, an Agilent Eclipse plus C18 column (2.1 × 50 mm, 1.8 µm) was used as stationary phase, and a gradient mixture of methanol (A) and 0.1% formic acid in water (B) as mobile phase. The gradient was: 0 min, 2% A; 0.75 min, 2% A; 11 min, 100% A; 13.5 min, 100% A; 14 min, 2% A and isocratic up to 15 min. Flow rate was 0.4 mL/min. MS parameters were as follows: sampling cone voltage, 40 V; source offset, 80 V; capillary voltage, 3500 V; nebulizer gas (N_2_) flow rate, 800 L/h; desolvation temperature, 450 °C. The mass accuracy and reproducibility were maintained by infusing lockmass (leucine-enkephalin, [M–H]^−^ = 554.2620 *m*/*z*) thorough Lockspray at a flow rate of 20 μL/min. Centroided data were collected in the *m*/*z* range 50 to 1200, and the *m*/*z* values were automatically corrected during acquisition using lockmass.

### 2.5. Determination of Antioxidant Capacity

By referring to Uysal et al. [28], the radical scavenging and reducing power abilities of the extracts were evaluated using ABTS, DPPH, CUPRAC, and FRAP. Likewise, ferrous ion chelating and phosphomolybdenum experiments were performed. The results were given as standard molecules equivalents of Trolox (TE) or ethylenediaminetetraacetic acid (EDTA), respectively.

### 2.6. Determination of Enzyme Inhibitory Effect

The ability of extracts to inhibit effects of a panel of key enzymes, such as cholinesterases, α-glucosidase, α-amylase, and tyrosinase were investigated according to the protocol described by Grochowski et al. [29]. The enzyme inhibitory activities of extracts were expressed as equivalents of acarbose for α-glucosidase, α-amylase, galatamine for cholinesterases, kojic acid for tyrosinase, and catechin for elastase.

### 2.7. Antimicrobial Activities

To determine antimicrobial activities of *P. reticulatus* extracts, microdilution method was performed as done in our earlier paper. In the experiments, Gram-positive (*Staphylococcus aureus* (ATCC 6538), *Listeria monocytogenes* (NCTC 7973), and *Bacillus cereus* (clinical isolate)) and Gram-negative (*Salmonella* Typhimurium (ATCC 13311), *Pseudomonas aeruginosa* (ATCC 27853), *Enterobacter cloacae* (human isolate), and *Escherichia coli* (ATCC 35210)) bacteria were used. The tested bacteria were obtained from the Institute for Biological Research “Sinisa Stankovic”, Belgrade, Serbia.

Regarding anti-fungal properties, different fungal strains were selected, and they were *Aspergillus fumigatus* (human isolate), *Aspergillus ochraceus* (ATCC 12066), *A. niger* (ATCC 6275), *A. versicolor* (ATCC 11730) can be considered as significant for aspergilloses. *Trichoderma viride* (IAM 5061), *Penicillium funiculosum* (ATCC 36839) are attacking plant and especially pineapples, *P. ochrochloron* (ATCC 9112), and *P. verrucosum* var. *cyclopium* (food isolate) are contaminant of foods, especially meat products.

Obtained results from antibacterial and antifungal assays were evaluated the minimum inhibitory concentration (MIC) and the minimum bactericidal/fungicidal concentration (MBC/MFC) values. In antibacterial activity, streptomycin and ampicillin were selected as standards (1 mg/mL). In antifungal activity, ketoconazole and bifonazole were selected as standards (0.10–0.35 mg/mL).

### 2.8. Computational Tests

#### 2.8.1. Receptor Preparation

The crystal structure of the enzyme tyrosinase used for the in silico tests has been downloaded from the Protein Data Bank RCSB PDB [30]; Tyrosinase, pdb id: 2Y9X, [31] in complex with tropolone. The raw structure has been polished with UCSF Chimera [32] by removing additional chain, and the B chain was used as crystallographic protein complex. The protein was prepared with the Protein Preparation Wizard [33] implemented in Maestro suite 11.1 [34], by filling missing side chain and loops e-converting seleno-methionines and seleno-cysteines to methionines and cysteines, respectively, generating heteroatoms states at pH 7.4 using Prime [35], using a well-established procedure previously reported by our research group [36,37,38,39] then deleting non-catalytic waters and other co-crystallized molecule, were conducted the H-bond assignment by using PROpKA at pH 7.4, and a minimization of only the hydrogens. The copper ions present in the catalytic pocket of tyrosinase, have been optimized by the automatic generation of optimized metal states. The protein was separated from the crystallographic ligand and used without further modifications for the docking experiments and self-docking validation process.

#### 2.8.2. Self-Docking Validation

In order to assess the suitability of the software GOLD 5.5 [40] for the docking on tyrosinase, a procedure of self-docking was performed as reported previously by Stefanucci et al. [41]. At this stage, the software GOLD 5.5 was configured for self-docking involving the crystallographic tyrosinase inhibition-enzyme complex chain B (2Y9X), as described above. All the scoring functions of GOLD (ASP, PLP, GOLDSCORE, and CHEMSCORE) were considered to conduct the validation tests, performing a self-docking of the crystallographic ligands, and by comparing the root-mean-square deviation (RMSD) of the best docked crystallographic pose with the original crystallographic pose. An area of 15 Å around the co-crystallized ligand was defined as the binding site. At the end of the docking calculations, the ChemScore and GoldScore scoring functions returned with the lowest RMSD value. Both ASP and PLP methods were not able to find any interactions for tropolone with the copper atoms, thus have been discarded [42]. Finally, both Goldscore and Chemscore were able to find a similar pose of tropolone superimposable to the crystallographic one with similar RMSD in the range of 2 to 3 angstroms. Considering that the GoldScore scoring function is defined as the most comprehensive scoring function embedded in GOLD docking software [43], this scoring function has been employed for the docking experiments in this work.

#### 2.8.3. Ligand Preparation

The selected compounds (Table 1) were prepared with LigPrep tool [44] embedded in Maestro 11.1, neutralized at pH 7.4 by Ionizer and minimized with OPLS-3 force field [45] and used for the docking experiment without further modifications.

### 2.9. Molecular Docking

**Figure 1:**
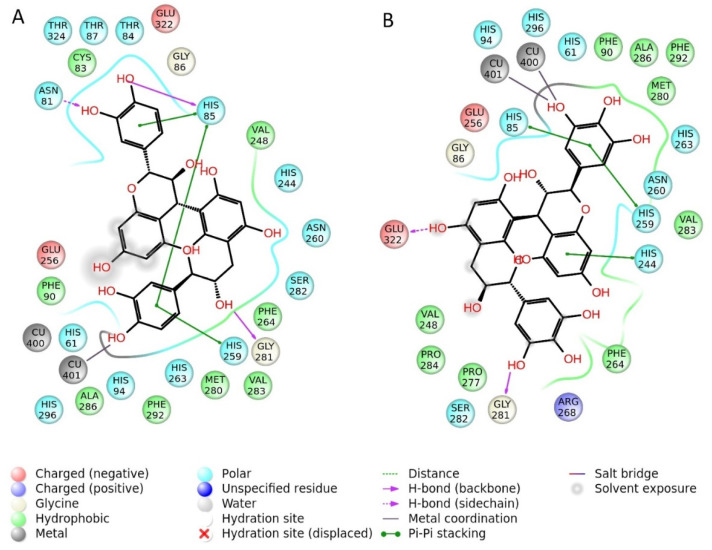
(**A**) Procyanidin B3; (**B**) Prodelphynidin B3 docked to tyrosinase.

Dockings of the selected molecules (see Table 1) were performed on tyrosinase by using the software Gold 5.5, developed by the Cambridge University [40]. GoldScore scoring function was employed for the docking calculations. The docking grid was determined by a radius of 15 Angstroms around the crystallographic ligand center, similarly to the previous published works [46,47,48]. As results of the docking experiments, the two substances with the highest Fitness Score “GoldScore” were Prodelphynidin B3 with a score of 68.18 and Procyanidin B3 with a score of 66.74. The best pose for these latter compounds docked to tyrosinase are depicted in Figure 1.

### 2.10. Data Analysis

A one-way analysis of variance was carried out to investigate significant differences in phytochemical contents, antioxidant, enzyme inhibitory, antifungal, and antibacterial activities in the different extracts (*p* < 0.05, Turkey’s post hoc test). The computation of Pearson’s correlation coefficients allowed to analyze the relationship between the phytochemical contents and the antioxidant and enzyme inhibitory activities.

Afterwards, the dataset was scaled and subjected to multi-block discriminant analysis for the purpose of investigating the variation of the evaluated biological activities between the two parts, as well as the two extraction solvents. The sensibility and specificity of the two models was evaluated by calculating the AUC value. R v 3.6.2 statistical software was used for the analysis.

## 3. Results

### 3.1. Chemical Composition

The concentration of total phenolic, flavonoids, phenolic acid, and flavanol compounds of the different extracts was reported in Table 2. The content of phenolic and flavonoid ranged between 125.67 to 131.83 mg GAE/g and between 0.63 to 44.95 mg RE/g, respectively. Similarly, that of phenolic acid and flavanol varied from 24.88 to 6.58 mg CAE/g and from 5.99 to 14.14 mg CE/g, respectively. Both total phenolic and flavanol contents were maximum in the methanol stem bark extract (131.83 ± 1.81 mg GAE/g and 14.14 ± 0.11 mg CE/g, respectively). By contrast, the content of flavonoids and phenolic acids of the water leaves extract were higher than that of the other extracts (44.95 ± 0.38 mg RE/g and 63.58 ± 2.00 mg CAE/g, respectively).

Thirty-two different phenolic constituents were identified and quantified combining the data of the Q-Tof for the high-resolution mass spectrometry and MS^n^ fragmentation pathways obtained using ion trap. Results are summarized in Table 3, and clearly revealed that both plant parts contain large amount of condensed tannins, mostly prodelphyinidine and procyanidins. Identified compounds were prodelphynidins, procyanidins [49,50], catechin and epicatechin, in bark, while flavonoids in leaves mostly myricetin, quercetin, and kaempferol derivatives [51,52,53]. Identification of compounds was obtained matching accurate mass measurements, fragmentation pathways, and literature [48,49,50,51,52], confirmation of some of the compound was also obtained by co-injection of reference standard. Considering phenolic constituents, the extraction with methanol for the plant part result more favorable. This result indicate a different extraction behavior of the flavonoid glycosides and procyanidin derivatives in water and in methanol, this result can be explained by the different solubility of the compounds in the two solvents, by the presence of other constituents that can interfere with the extraction and that are not measured, such as sugars, olygosaccharides, as well as lipid and waxes, as well as due to the different ability of the water and methanol to penetrate dried plant material.

### 3.2. In Vitro Antioxidant Properties and Enzyme Inhibitory Activities

Six different in vitro assays including ABTS and DPPH radical scavenging, Ferric and cupric ion reducing, phosphomolybdenum and ferrous ion chelating were used to assess the antioxidant capacity of the prepared extracts. Overall, the antioxidant capacity of methanol stem bark extract was significantly high than that of the other extracts (Table 4). In depth, these activities were enumerated as follows: DPPH: 1.94 ± 0.01 mmol TE/g, ABTS: 3.31 ± 0.01 mmol TE/g, FRAP: 2.86 ± 0.02 mmol TE/g, CUPRAC: 5.09 ± 0.08 mmol TE/g, phosphomolybdenum: 5.16 ± 0.23 mmol TE/g, and metal chelating: 17.12 ± 0.46 mg EDTAE/g. In addition, as seen in DPPH assay, methanol leaves extract also exhibited highest activity.

The inhibitory effects of the extracts against the six key enzymes were shown in Table 4. Among the extract, methanol stem bark exhibited a potent anti-AChE and anti-BChE activities, being 2.54 and 5.48 mg of galatamine acid equivalent/g of sample for AChE and BChE, respectively. Moreover, the anti-tyrosinase activity increase in the following order: leaves-methanol > stem bark-methanol > leaves-water > stem bark-water. Remarkably, except methanol leaves, it was found that all extracts possessed the same anti-elastase activity (leaves-water: 1.98 ± 0.10 mmol CE/g, stem bark-water: 2.08 ± 0.06 mmol CE/g, stem bark-methanol: 2.21 ± 0.12 mmol CE/g). Concerning both anti-hyperglycemia assays, water leaves, and methanol leaves extracts had the strongest anti-amylase (0.97 ± 0.01 mmol ACAE/g) and anti-glucosidase (0.97 ± 0.01 mmol ACAE/g) activities, respectively. No anti-glucosidase activity was observed in water extracts of both studied parts.

**Figure 2:**
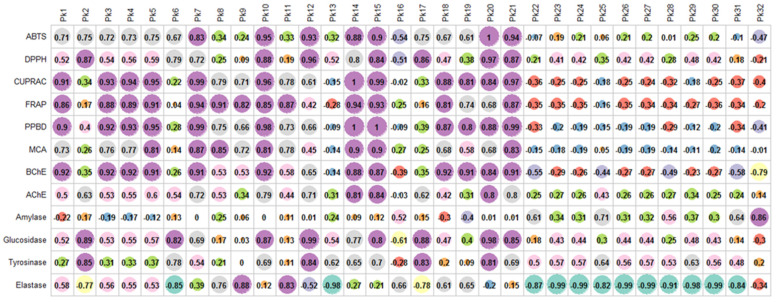
Relationship between phytochemical compounds and antioxidant and six key enzyme inhibitory activities.

Afterward, the contribution of the phytochemical compounds to the antioxidant and enzymes inhibitory activity was determined. As shown in Figure 2, the antioxidant activity and metal chelating ability of *P. kotschyi* extracts seemed to be link to several phytochemical compounds by considering the Pearson’s coefficient being higher than 0.8. Similarly, both anti-cholinesterase, anti-glucosidase, anti-tyrosinase, and anti-elastase activities were bound with more than one compounds. Interestingly, Pk10, Pk14, Pk15, and Pk21 were significant positive correlation with all of the antioxidant activities (ABTS, DPPH, CUPRAC, FRAP, PPBD, and MCA), as well as anti-cholinesterase activities. Significant correlation was obtained between Amylase and a single compound (Pk32).

### 3.3. Antifungal and Antibacterial Activities

The antifungal and antibacterial effects of the extracts on the tested eight fungal strains are depicted in Table 5 and Table 6, respectively. The different extracts showed, overall, remarkable fungistatic and fungicidal, as well as bacteriostatic and bactericidal effects to variable extents, depending on the strain in question. Concerning the fungistatic effect, the results revealed that *A. versicolor* was more sensitive to leaves water and bifonazole, with a MIC value of 0.10 mg/mL. *A. ochraceus* was more susceptible to water and methanol extract of leaves (MIC = 0.10 mg/mL) as compared to remaining extracts and reference drugs. Though our mind that both extracts of stem bark (MIC = 0.15 mg/mL) were found to be more effective towards the two above mentioned strains (*A. versicolor* and *A. ochraceus*) than ketoconazole (MIC = 0.20 mg/mL). In the case of *Aspregillus fumigatus*, stem bark water had the lowest MIC value (0.11 mg/mL), while the MIC value of stem bark methanol and Bifonazole were equal (0.15 mg/mL). The lowest MIC value for *Penicillium ochrochloron* was obtained with water extracts of the two part (0.15 mg/mL). Both leaves extracts and stem bark water exhibited the best effectiveness toward *P. funiculosum* and *T. viride* than the two reference drugs. In addition, stem bark methanol and bifonazole had similar minimum inhibitory concentration for *T. viride* (0.15 mg/mL). The efficiency of water and methanol extracts of leaves against *A. niger* were comparable to that of ketoconazole (0.15 mg/mL); similarly, leaves water, methanol stem bark, water stem bark, and ketoconazole showed similar MIC value for *P. verrucosum var. cyclopium*. Regarding the fungicidal effect, some tested extracts effectively eliminate studied strains, at different concentrations, some of which were comparable to that of the controls. The best minimum fungicidal concentration (MFC) values were observed for *A. fumigatus*, *P. ochrochloron*, *P. funiculosum,* and *T. viride*. Indeed, *A. fumigatus* and *T. viride* was eliminated by stem bark water at 0.15 mg/mL. 0.21 mg/mL of leaves water allowed to kill *P. ochrochloron* while 0.20 mg/mL of leaves methanol and 0.21 mg/mL of leaves water were received sufficient to eliminate *P. funiculosum*. These concentrations were lower than the two reference drugs.

The best bacteriostatic and bactericidal effects for most of the studied bacterial strains was recorded by stem bark methanol. In depth stem bark methanol inhibit growth of *M. flavus*, *S. aureus*, *L. monocytogenes*, *E. coli*, *P. aeruginosa*, *E. cloacae*, and *S. typhimurium* at MIC value of 0.04, 0.04, 0.04, 0.11, 0.11, 0.03, 0.07 mg/mL, respectively. Similarly, *M. flavus*, *S. aureus*, *L. monocytogenes*, *E. coli*, *P. aeruginosa*, *E. cloacae* and *S. typhimurium* were found to be sensitive to stem bark methanol at MBC value of 0.07, 0.08, 0.08, 0.15, 0.16, 0.07, and 0.15, respectively. Otherwise, stem bark water exhibited excellent bacteriostatic and bactericidal activities against *S. aureus* (MIC = 0.03 mg/mL; MBC = 0.07 mg/mL), while leaves methanol remarkable bacteriostatic and bactericidal activities effect toward Listeria monocytogenes (MIC = 0.05 mg/mL, MBC = 0.20 mg/mL). It should be noted that the MIC and MBC values of above mentioned extracts were lower than the two controls. Additionally, the efficiency of some extracts to inhibit the growing or to kill several strains was comparable to one or both reference drugs; illustratively, leaves methanol-ampicillin-streptomycin showed equal MIC value for *S. aureus* (0.10 mg/mL) while leaves methanol- streptomycin had similar MBC value for the same strain (0.20 mg/mL). The same trend was observed with stem bark water and ampicillin for *E. coli* (MIC = 0.15 mg/mL), stem bark water, and both controls (MBC = 0.15 mg/mL) and those are just a few examples.

**Figure 3:**
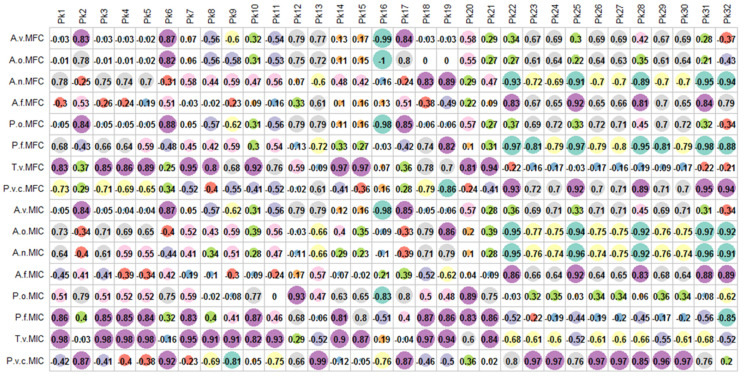
Relationship between phytochemical compounds and antifungal activities.

**Figure 4:**
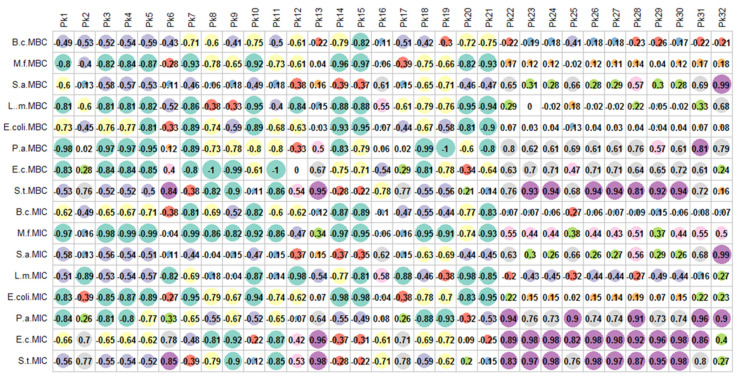
Relationship between phytochemical compounds and antibacterial activities.

Thereafter, the relationship between phytochemical compounds and the evaluated antifungal and antibacterial activities was investigated. As seen from results (Figure 3 and Figure 4), the MIC and MFC activities of various fungal strains seemed due to the effect of multiple compounds (Pearson’s coefficient being higher than 0.8). Remarkably, the MFC and MIC of T. *viride*, the MIC of *P. funiculosum* and *P. verrucosum var. cyclopium* had significant positive correlation with a largest number of compounds. Contrary to anti-fungal activity, vary few significant positive correlation was found between anti-bacterial activities and chemical compounds. However, the bacteriostatic and bactericidal activities of *S. typhimurium,* as well as the bacteriostatic activity of *E. coli* seemed result from the action of a large number of chemical compounds.

### 3.4. Antioxidant, Enzyme Inhibitory, Antifungal, and Antibacterial Activities Variation among Extraction Solvents and Plant Parts

**Figure 5:**
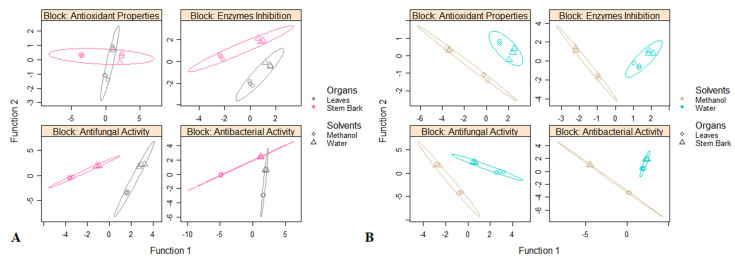
Score plot of the multi-block discriminant analysis on *P. kotschyi* bioactive antioxidant, enzyme inhibitory, antifungal, and antibacterial activities. (**A**): Discrimination of the samples according to the parts. (**B**): Discrimination of the samples according to the solvents of extraction.

**Figure 6:**
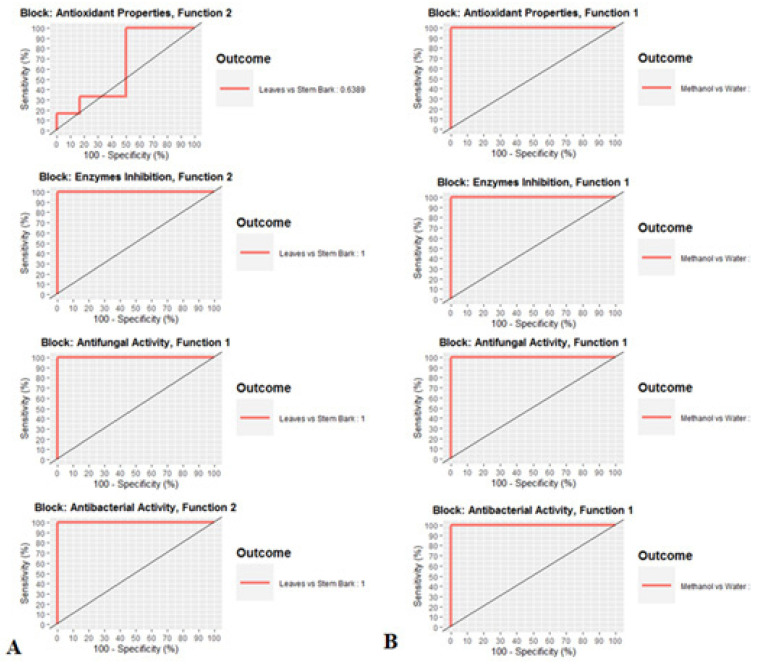
Goodness of the models assessment by Area Under the Curve average using one-vs-all comparisons. (**A**): model 1 (Leaves versus Stem bark). (**B**): model 2 (Methanol versus Water).

The results of multi-block discriminant analysis considering parts and extraction solvents as class membership criteria were graphically shown in Figure 5A. looking at the both scatterplots of samples, it is possible to view a quite clear separation among the two parts and extraction solvents, respectively, except the block “Antioxidant Properties” wherein no segregation between the leaves and stem bark was obtained (Figure 5A,B). For the first model (parts), 2 functions, were sufficient to discriminate the two parts for the block “Enzymes Inhibition” and “Antibacterial Activity” while 1 functions, was sufficient to separate the two parts for the block “Antifungal Activity” (Figure 6A). Regarding the block “Antioxidant Properties”, an AUC value of 0.63 was obtained considering 2 functions, suggesting no significant difference between both parts as regards the antioxidant activities (Figure 6A). Concerning the second model (extraction solvent), the optimal number of functions was found to be 1 for the all the blocks (Figure 6B).

### 3.5. Molecular Docking

A series of ten bioactive natural compounds, found in the extracts of *P. kotschyi* have been submitted to in silico docking experiments in order to evaluate their affinity for the enzymatic cavity of the enzyme tyrosinase. This enzyme was selected among the other tested ones due to the relevant anti-tyrosinase activity showed by the *P. kotschyi* extracts. Two substances, namely Procyanidine B3 and Prodelphynidin B3 have been found to have the higher Fitness score calculated by the scoring function GoldScore embedded in the software GOLD 5.5. The scoring function GoldScore ranks the docking results taking in consideration several parameters, it has been optimized for the prediction of ligand binding positions and takes into account factors such as H-bonding energy, van der Waals energy, metal interaction, and ligand torsion strain [54]. From the analysis of the best docking pose of procyanidin B3 it can be observed that the ligand-enzyme complex is stabilized by three hydrogen bonds respectively with Asn81, His85, and Gly280, also the molecule is able to form several π-π stacks to the aromatic side chains of His85, His244, His259. Additionally, the phenolic hydroxyl group of the Procyanidin B3 is able to form a coordination bond to Cu401, directly involved in the mechanism of the enzyme. The other best ranked substance was Prodelphinidin B3. The ligand-enzyme complex with the best pose obtained, is stabilized by two hydrogen bonds, respectively, toward Glu322 and Gly281, several π-π stacks with His85, His244, and His259. Additionally, this molecule was able to establish two coordinative bonds to both the copper atoms present in the enzymatic cavity.

## 4. Discussion

**Figure 7:**
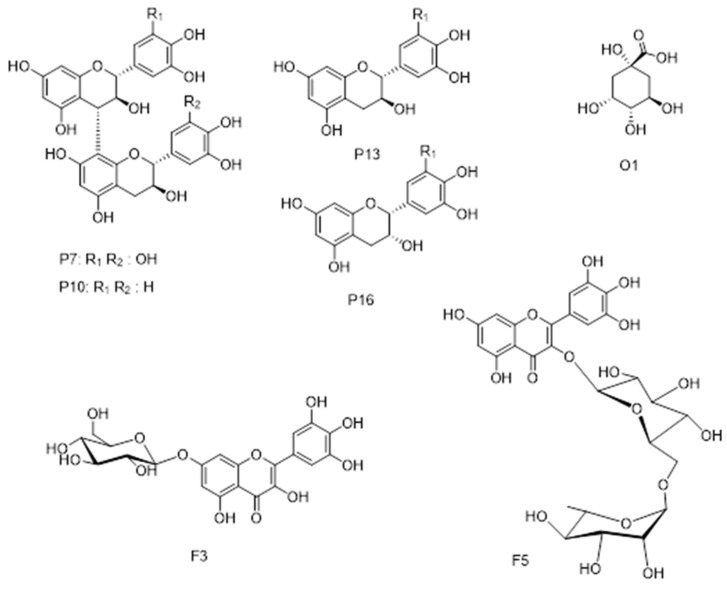
Main components in *P. kotschyi* extracts.

The antioxidant property of extracts derived from the leaves and stem bark of *P. kotschyi* was determined by different methods based on different mechanisms. In fact, because no single method can accurately reflects the mechanism of action of all antioxidant molecules contained in a plant extract, Tabart et al. [55] recommend the necessary achievement of different assays at once. Based on the experimental outcomes, the high antioxidant capacity was recorded with methanol stem bark extract, whatever the assay performed. A previous several in vitro studies on different extracts of *P. kotschyi* disclosed it potential antioxidant activity [16,56,57]. Apart from in vitro assays, the excellent antioxidant property of *P. kotschyi* was also demonstrated by various in vivo experiments. Indeed, Eleha et al. [58] reported that water leaves and methanol bark extracts of *P. kotschyi* possesses anti-oxidative effect, comparable to that of vitamin C, against paracetamol induced liver damage of rats. In addition, 48 hourly administration of the aqueous extract of *P. kotschyi* to pentylenetetrazole-induced kindled rats, allowed to reduce the progression of epileptogenesis through inhibition of oxidative stress [56]. Furthermore through LC-MS^n^ analysis, several well-known antioxidant molecules were identified in the different extracts. The presence of prodelphynidin derivatives in large amount in leaves and bark extract, as well as epicatechin and catechin (Figure 7) can explain the observed antioxidant activity. Strong antioxidant activity is associated with the presence of two or three hydroxyl substituents in the “B” ring of the flavonoid skeleton [59,60,61,62]. Myricetin and quercetin derivatives also can contribute to the antioxidant activity of the leaves extract samples. We studied antioxidant activity of isolated kaempferol and quercetin glycosidic derivatives showing very high activities for quercetin-3-O-rhamnoside and rutin compared to Kempferol-7-O-β-glucopyranoside-3-O-rutinoside [63], as also confirmed by previous investigations comparing kaempferol and quercetin derivatives [62]. Based on the above findings, *P. kotschyi* leaves and stem bark appears clearly as a valuable source of antioxidant compounds that could replace synthetic antioxidants, such as tertiary butyl hydroquinone, butylated hydroxyanisole and butylated hydroxytoluene, since there have been reported to have health risk and toxicity [64].

Past few years, oxidative stress-related disorders caused by reactive oxygen species (ROS) have deserve special attention. In fact, ROS are generated as a natural by-product during the normal metabolism processes of oxygen and contributes to the protection for living organisms. Nevertheless, excessive production of ROS is detrimental to the body and result in oxidative stress which is involved in pathogenesis of several chronic disorders [65,66,67]. Disorders, such as neurodegenerative, cancer, cardiovascular, diabetes mellitus, are among the most frequent. Several studies have been done in order to determine the subjacent triggering factors, in particular to understand the role of free radicals in the occurrence these disorders, and also to discover potential substances towards preventing the occurrence of oxidative damages [68]. As part of anti-ROS agents, antioxidant, from both synthetic and natural sources, have proved to be efficient to regulate the production of free radicals, to minimize its undesirable effects and, hence, to prevent a wide variety of above mentioned human disorders [69]. As observed above *P. kotschyi* leaves and stem bark proved to be an excellent source of antioxidant compounds, which could be potent enzyme inhibitory agents against various enzymes involve in non-communicable disorders including diabetes mellitus, Alzheimer’s disease, and skin’s disorders. Thereby, the extract of both parts were screened for their possible anti-alpha-amylase, anti-alpha-glucosidase, anti-cholinesterases, anti-tyrosinase, and anti-elastase effects.

In view of the results, methanol stem bark exhibited the highest anti-cholinesterases activities. Phenolic acid and flavanol compounds contributed significantly to the observed inhibition effects. Subsequent surveys have determined the relevance of both BChE and AchE in the pathophysiology of Alzheimer’s disease and have demonstrated the therapeutic benefit of the inhibition of both BchE and AchE. In fact, it has been reported that AchE activity is reduced by 67% compared to the normal level in the hippocampus and temporal lobe during the progression of Alzheimer’s disease while the BchE activity is gone up to 165%, relative to normal levels [70]. This imbalance in the AChE/BChE ration causes acetylcholine deficit in the brain. Thus, AChE and BChE inhibitors prevent hydrolyze of the acetylcholine and, thereby, improving the deficient cholinergic neurotransmission. Flavonoid derivative have been considered as significant AChE inhibitors (Khan 2018). A few phenolic compound present in *P. kotschyi* extracts are reported to be potent inhibitors of AchE and BchE. For example, Khan in review indicate that activity may also be improved by increasing the gallation of catechin [71], and recently catechin of green tea have been considered for their Acetyl- and Butyryl-Cholinesterase Inhibition activity sowing that the most promising compound is epigallocatechin gallate [72].

Related to anti-hyperglycemia assays, water leaves extract had the strongest anti-amylase activity while methanol leaves extract showed the highest anti-glucosidase activities. In an experiment carried out on alloxan induced diabetic rats, the reduction in the blood glucose levels of the rats after four days of treatment with *P. kotschyi* was demonstrated [12]. Another study dealing with the analysis of the biochemical and hematological parameters in alloxan-induced diabetic rats treated with *P. kotschyi* roots, result on the decrease in blood urea, creatinine, glucose levels, ALT, and AST [13]. From a significant increase in the leukocyte and erythrocyte counts, PCV and Hb values were observed [13]. All these results may explain the traditional use of the plant by traditional healers as anti-diabetes mellitus agents [12]. Furthermore, the presence of proanthocyanidin groups in the extracts provides further evidence for the observed anti-amylase and glucosidase effects. [73] reported that proanthocyanidins significantly ameliorate the high blood sugar, the hyperlipidemia and the oxidative stress in the mouse model of type 2 diabetes. Although treatment of insulin-resistant mice with proanthocyanidin-rich extract derived from longan flower enhanced the expression of glucose transporter 4 and insulin receptor substrat-1 and declined the systolic blood pressure [74].

Additionally, leaves-methanol extract which exhibited the highest tyrosinase inhibitory activity, showed no inhibitory activity with elastase. However, the remaining extracts inhibited elastase without any significance difference between them. The development of new effective cosmetic products from nature to improve the appearance of the skin and delaying premature aging significantly bounced back over the years. In fact, natural-called cosmetics products are very fashionable for consumers throughout the world. This can be explained by the fact that numerous research has shown that herbals contain biomolecules, having a significant effect on skin brightening similarly to sunscreen and skin aging as antioxidant agents. The inhibitors of elastase are useful to overcome the loss of skin sagging and elasticity, while those of tyrosinase allow to limit melanin synthesis and, hence, ensure a breakdown of skin disorders, i.e., hyperpigmentation and melanoma [75]. In this regard, inhibition of elastase and tyrosinase enzymes are considered as a significant targets in cosmetic industry for researches to find various kind of new skin brightening and aging agents. LC-MS^n^ analysis showed that *P. kotschyi* contained several potential anti-tyrosinase and anti-elastase compounds, in particular proanthocyanidins that could retard the aging and abnormal pigmentation processes, by acting as strong H-donors, oxygen quenchers, or reducing agents [76]. Therefore, *P. kotschyi* can be used in various sectors of the cosmetic industry as natural additives ingredients.

Foodborne disease is a serious medical issue. Indeed, various well-publicized outbreaks of foodborne acute or chronic disease have alerted researcher’s awareness that adverse microorganisms may be present in food, at the origin of these diseases. In addition to acute gastrointestinal issues, certain foodborne pathogens, through complex interactions with the human immune response, cause diseases that impact various major organ systems [77]. Given that foodborne disease is a main cause of morbidity and mortality, the suppression of causative pathogens presents a unique challenge for scientists, since the emergence of multi-drug resistance microorganisms. Indeed, fungi and bacteria constantly develop mechanisms of resistance to currently available drugs. The discovery of novel and effective antimicrobial drugs is the best approach to overcome fungal and bacterial resistance and develop appropriate new food preservatives or new effective therapeutic agents to combat human infectious diseases caused by microorganisms. For this purpose, the antimicrobial activity of secondary metabolites occurring in medicinal plants has been intensively investigated towards a wide range of fungi and bacteria. In this study, leaves and stem bark extracts of *P. kotschyi* showed promising antifungal and antibacterial activity. Far better, a few extracts displayed more pronounced antifungal and antibacterial activity than the reference drug used in this study. Illustratively, an interesting fungistatic or fungicidal effect towards *A. ochraceus*, *A. fumigatus*, *P. ochrochloron*, *T. viride* and *P. funiculosum* was observed with a MIC and MFC value ranging from 0.10 to 0.11 mg/mL and 0.15 to 0.21 mg/mL, respectively. These findings justify, for the first time, the possibility of using the extracts of *P. kotschyi* stem bark and leaves for preserving food against spoilage microorganisms or for managing human infections caused by said pathogens. Similarly a good bacteriostatic or bactericidal was observed against *M. flavus*, *S. aureus*, *L. monocytogenes*, *E. coli*, *P. aeruginosa*, *E. cloacae,* and *S. typhimurium* with a MIC and MBC value varying from 0.3 to 0.7 mg/mL and 0.7 to 0.21 mg/mL, respectively. Alain et al. [78] reported that *E. coli* had bacteriostatic and bactericidal sensitivity with ethanolic extract of *P. kotschyi* stem bark at concentration of 0.39 and 0.78 mg/mL, respectively. Additionally, the hydroethanolic extract of stem bark exhibited the highest bacteriostatic and bactericidal effect on *S. aureus* with MIC and MBC of 0.39 and 0.78 mg/mL, respectively [78]. Similarly, the ethyl acetate extract of *P. kotschyi* leaves was reported to be active against *S. aureus*, *E. coli* and *S. pyogens* at MIC value of 10 mg/mL [79]. These findings and reports could explain why the root of the *P. kotschyi* is commonly used as a toothpick for oral hygiene, since it proved to be active towards two enteropathogenic Gram-positive bacteria, i.e., *S. auricularis* and *S. aureus* [80]. The antifungal and antibacterial displayed by the studied extracts may be due to the presence of several molecules highlighted in their phytochemical screening. Indeed, the extracts were rich in variety of molecules of antimicrobial proprieties notably proanthocyanidins [81,82]. Mechanisms of action including cell membrane disruption, DNA gyrase and protein konase inhibition, dehydratase inactivation (HpFabZ) and type III secretion inactivation, which contribute to the antibacterial activity of mostly derived plant molecules have been reported [83]. Similarly, plant derived molecules act as antifungal agent by inhibiting the biofilm formation and the morphogenetic switch, activating the apoptotic pathway, increasing reactive oxygen species levels and interfering with the synthesis of different components (i.e., glucan, ergosterol, chitin, glucosamine, and proteins) in fungi [84]. Thereby, it can be said that not only *P. kotschyi* leaves and stem bark extracts can be used as antimicrobial agents but also in synergism with the commonly used antimicrobial drugs in order to potentiate their efficiency and to decrease synthetic antibiotic dose and hence to reduce their adverse reactions.

The solubility of the bioactive compounds and, in turn, the bioactivities were mostly impacted by the nature of the solvent used. These findings agree with earlier studies reporting the influence of solvents on the nature and content of molecules and their biological activities [85]. Overall, the maximum presence of bioactive compounds, as well as most of the better bioactivities were obtained with the methanol. Nevertheless, bearing in mind the toxicity of methanol, it would be recommender to aqueous solvent, since it contain numerous valuable molecules which have also shown excellent activities. On the other hand, variation on bioactive compounds and bioactivities were observed among the stem bark and leaves. The stem bark appeared to be the part in which pharmaceutical properties is most concentrated. A similar result was obtained by [86], who found that stem bark of *Khaya senegalensis* was superior source of secondary metabolites and lead excellent pharmaceutical properties, compared with the leaves. These outcomes indicate that the difference of pharmaceutical properties observed between samples *P. kotschyi* depended not only on the difference in the polarity of extraction solvents but also on the part types. Further, the excellent pharmaceutical properties obtained with both polar solvents confirms the richness of this herbal in polar molecules.

## 5. Conclusions

In the current study, the water and methanol extracts of leaves and stem bark from *P. kotschyi* was investigated for their chemical composition and the antioxidant property. Likewise, the capacity to inhibit cholinesterase, amylase, glucosidase, tyrosinase, and elastase activity, as well as the antimicrobial activities were evaluated. LC-MS analysis allowed to identify thirty-two compounds in the tested extracts, prodelphinidin derivative was the most abundant in stem bark, while epicatechin the most abundant in leaves. As far as the bioactivities, the extracts showed remarkable antioxidant properties and promising outcomes for the management of hyperglycemia, Alzheimer’s disease, and skin disorders. The extracts also show the antifungal and antibacterial effects on different microorganism strains. Afterward, it was evidence that the solubility of bioactive compounds and the bioactivities were depended on the extraction solvents and the plant parts. These antioxidant properties, enzymes inhibitory capacities, and antimicrobial activities potential could be, at least in part, ascribed to the phenolic constituents revealed in the extracts by LC-MS analysis. Finally, obtained data suggest that *P. kotschyi* could be used to promote human health on the understanding that further research regarding it toxicity be assessed.

## Figures and Tables

**Table 1:** Docking scores expressed as Gold Fitness Score.

Compound Name	GOLD Fitness Score (Gold Score)
Epigallocatechin	54.83
Prodelphynidin B3	68.18
Procyanidin B2	52.04
Procyanidin B3	66.74
Catechin	58.02
Epicatechin	54.97
Quinic acid	50.97
Myricetin-7-glucoside	57.64
Myricetin-3-rutinoside	53.46
Quercetin-3-O-rutinoside	55.53

**Table 2:** Quantitative phytochemical determinations of *P. kotschyi* leaves and stem bark extracts *.

	*p*-Value	Leaves Methanol	Leaves Water	Stem Bark Methanol	Stem Bark Water
Total phenolic content (mg GAE/g)	0.0001	127.18 ± 1.85 ^b^	126.81 ± 0.89 ^b^	131.83 ± 1.81 ^a^	125.67 ± 0.89 ^b^
Total flavonoid content (mg RE/g)	0.0001	43.79 ± 0.30 ^b^	44.95 ± 0.38 ^a^	0.63 ± 0.17 ^d^	3.30 ± 0.14 ^c^
Total phenolic acids content (mg CAE/g)	0.0001	24.88 ± 0.58 ^c^	63.58 ± 2.00 ^a^	54.44 ± 2.91 ^b^	53.76 ± 4.01 ^b^
Total flavanols content (mg CE/g)	0.0001	7.77 ± 0.06 ^b^	5.99 ± 0.05 ^d^	14.14 ± 0.11 ^a^	6.24 ± 0.01 ^c^

* Values are reported as mean ± S.D. GAE: Gallic acid equivalent; RE: Rutin equivalent; CAE: Caffeic acid equivalent; CE: Catechin equivalent. a–d line wise values with same superscripts of this type indicate no significant difference among extracts (*p* > 0.05).

**Table 3:** Identified and quantified constituents of *P. kotschyi* leaves and stem bark extracts using LC-DAD-MS^n^ (mg/g), * indicate confirmed identification by reference compound comparison. Number indicating compounds are the one used for the structure of the main compounds reported in Chart 1 and of LC-MS chromatograms of Chart 2.

Retention Time (min)	Identification	[M−H]^−^ HR-MS	Molecular Formula (Exact Mass)	Fragments	Leaves Methanol	Leaves Water	Stem Bark Methanol	Stem Bark Water
2.3	P1 prodelphynidin gallocatechin-gallocatechin	609.1263	C30H25O14 (609.1244)	441 423 305 297 255	n.d.	n.d.	0.74 ± 0.02	0.15 ± 0.01
2.5	P2 prodelphynidin B3 isomer 1	593.1341	C30H25O13 (593.1295)	467 441 425 407 289	1.55 ± 0.01	0.36 ± 0.01	0.94 ± 0.02	0.27 ± 0.01
3.6	P3 prodelphynidin gallocatechin-gallocatechin	609.1265	C30H25O14 (609.1244)	441 423 305 297 255	n.d.	n.d.	0.47 ± 0.01	0.08 ± 0.01
5.2	P4 prodelphynidin B3 isomer 2	593.1315	C30H25O13 (593.1295)	467 441 425 407 289	n.d.	n.d.	1.06 ± 0.01	0.16 ± 0.01
5.7	P5 prodelphynidin gallocatechin-gallocatechin	609.1274	C30H25O14 (609.1244)	441 423 305 297 255	n.d.	n.d.	0.97 ± 0.01	0.09 ± 0.01
6.6	P6 epigallocatechin *	305.0715	C15H13O7 (305.0661)	261 219 218 179 165 125	3.32 ± 0.02	0.71 ± 0.01	1.69 ± 0.02	0.63 ± 0.01
6.7	P7 prodelphynidin B3 isomer 3	593.132	C30H25O13 (593.1295)	467 441 425 407 289	1.63 ± 0.01	0.77 ± 0.02	9.20 ± 0.02	0.60 ± 0.01
7.0	P8 prodelphinidin B3 isomer 4	593.132	C30H25O13 (593.1295)	467 441 425 407 289	n.d.	0.77 ± 0.01	1.35 ± 0.02	0.31 ± 0.01
7.3	P9 prodelphinidin B3	593.1332	C30H25O13 (593.1295)	467 441 425 407 289	n.d.	0.77 ± 0.02	1.32 ± 0.02	0.57 ± 0.01
7.7	P10 procyanidin B2 *	577.11394	C30H25O12 (577.1346)	451 425 407 289	2.08 ± 0.01	1.22 ± 0.02	3.46 ± 0.02	1.26 ± 0.02
7.9	P11 gallocatechin	305.0742	C15H13O7 (305.0661)	261 219 218 179 165 125	n.d.	0.71 ± 0.01	1.34 ± 0.01	0.45 ± 0.01
8.3	P12 procyanidin B dimer	577.1407	C30H25O12 (577.1346)	452 425 407 289	3.57 ± 0.02	1.45 ± 0.02	3.28 ± 0.02	1.62 ± 0.02
8.6	P13 catechin *	289.0761	C15H13O6(289.0712)		-	-	0.12 ± 0.02	0.21 ± 0.01
9.7	P14 procyanidin C1 isomer	865.2001	C45H37O18 (865.1980)	739 713 696 577 451 407	0.18 ± 0.02	0.15 ± 0.03	0.32 ± 0.02	0.18 ± 0.01
8.5	P15 procyanidin C1 isomer	865.1995	C45H37O18 (865.1980)	739 713 696 577 451 407	0.18 ± 0.02	0.13 ± 0.02	0.26 ± 0.02	0.19 ± 0.02
9.4	P16 epicatechin *	289.0777	C15H13O6 (289.0712)	245 205 179	14.78 ± 0.02	3.08 ± 0.02	1.48 ± 0.02	0.10 ± 0.01
9.4	P17 galloyl procyanidin B2	729.1489	C37H29O16 (729.1456)	245 205 179	1.47 ± 0.02	0.47 ± 0.01	0.95 ± 0.02	0.11 ± 0.01
11.8	P18 catechin gallate	441.0823	C22H17O10 (441.0821)	577 425 407 289	nd	nd	0.37 ± 0.02	0.09 ± 0.01
12.9	P19 acetyl Procyanidin dimer	635.1358	C32H27O14 (635.1401)	576 467 423	n.d.	n.d.	0.28 ± 0.01	
				total	28.7	13.7	31.0	9.1
2.1	O1 quinic acid *	191.0556	C7H11O6 (191.0556)	173 127 111 93 85	3.07 ± 0.02	3.40 ± 0.01	4.18 ± 0.02	4.57 ± 0.01
3.3	O2 galloyl glucoside	331.0649	C13H15O10 (331.0665)	271 211 169 125	0.11 ± 0.01	0.02 ± 0.01	0.27 ± 0.01	0.02 ± 0.01
		-		total	3.18	3.42	4.45	4.59
9.9	F1 myricetin-7-O-galloyl glucoside	631.09351	C28H23O17 (631.0935)	479 326 287 271 179	0.31 ± 0.01	0.20 ± 0.01	n.d.	n.d.
10.0	F2 myrecitin-3-O-rutinoside *	625.1385	C27H29O17 (625.1405)	420 316	0.31 ± 0.01	0.08 ± 0.01	0.01 ± 0.01	n.d.
10.2	F3 myrecitin-7-O-glucoside *	479.0819	C21H19O13 (479.0826)	316 271 179	4.24 ± 0.02	0.96 ± 0.02	0.03 ± 0.01	n.d.
10.8	F4 quercetin-3-O-glucoside gallate	615.0986	C28H23O16 (615.0986)	463 301 271 255 179 152	0.27 ± 0.01	0.19 ± 0.01	0.06 ± 0.01	n.d.
11.1	F5 myricetin-3-O-rutinoside	463.0876	C21H19O12 (463.0872)	316 287 271 179	8.92 ± 0.02	2.10 ± 0.02	0.12 ± 0.01	0.08 ± 0.01
12.3	F6 quercetin-3-O-rutinoside *	447.0925	C21H19O11 (447.0927)	301 271 179 151	3.47 ± 0.02	0.84 ± 0.02	0.05 ± 0.01	n.d.
12.7	F7 myricetin-rhamnoside-glucoside	625.1399	C27H29O17 (625.1405)	479 316 271	0.25 ± 0.01	0.14 ± 0.01	0.01 ± 0.01	n.d.
13.0	F8 kaempferol-3-O-rhamnoside *	431.0958	C21H19O10 (431.0978)	285 267 229	0.14 ± 0.03	0.04 ± 0.01	0.01 ± 0.01	n.d.
13.2	F9 methoxy-quercetin	317.03105	C15H9O8 (317.0297)	299 179 151	0.49 ± 0.02	0.11 ± 0.01	n.d.	n.d.
13.6	F10 quercetin-3-rhamnosyl-7-glucoside	609.1449	C27H29O17 (609.1456)	463 301 151	0.21 ± 0.04	0.14 ± 0.04	n.d.	n.d.
15.0	F11 quercetin *	301.041	C15H9O7 (301.3482)	179 151	0.43 ± 0.01	0.44 ± 0.02	n.d.	n.d.
				total	18.83	5.24	0.29	0.08

nd: not detected.

**Table 4:** Antioxidant and enzyme inhibitory properties of *P. kotschyi* extracts *.

		*p*-Value	Leaves Methanol	Leaves Water	Stem Bark Methanol	Stem Bark Water
Antioxidant assays	ABTS (mmol TE/g)	0.0001	2.95 ± 0.02 ^b^	2.15 ± 0.08 ^d^	3.31 ± 0.01 ^a^	2.28 ± 0.04 ^c^
	DPPH (mmol TE/g)	0.0001	1.91 ± 0.01 ^a^	1.65 ± 0.03 ^b^	1.94 ± 0.01 ^a^	1.59 ± 0.08 ^b^
	CUPRAC (mmol TE/g)	0.0001	3.59 ± 0.07 ^b^	3.43 ± 0.02 ^c^	5.09 ± 0.08 ^a^	3.14 ± 0.01 ^d^
	FRAP (mmol TE/g)	0.0001	1.86 ± 0.04 ^c^	2.11 ± 0.01 ^b^	2.86 ± 0.02 ^a^	1.66 ± 0.01 ^d^
	Phosphomolybdenum (mmol TE/g)	0.0001	3.78 ± 0.23 ^b^	3.48 ± 0.04 ^bc^	5.16 ± 0.23 ^a^	3.25 ± 0.09 ^c^
	Metal chelating (mg EDTAE/g)	0.0001	9.28 ± 0.44 ^c^	11.56 ± 0.07 ^b^	17.12 ± 0.46 ^a^	5.36 ± 0.34 ^d^
Enzyme inhibitory assays	AChE inhibition (mg GALAE/g)	0.0001	2.36 ± 0.02 ^b^	2.27 ± 0.03 ^c^	2.54 ± 0.02 ^a^	1.90 ± 0.04 ^d^
	BChE inhibition (mg GALAE/g)	0.0001	3.46 ± 0.16 ^b^	2.18 ± 0.25 ^c^	5.48 ± 0.10 ^a^	3.47 ± 0.25 ^b^
	Tyrosinase inhibition (mg KAE/g)	0.0001	77.39 ± 0.21 ^a^	68.32 ± 0.18 ^c^	75.93 ± 0.68 ^b^	57.32 ± 0.38 ^d^
	Amylase inhibition (mmol ACAE/g)	0.0001	0.72 ± 0.01 ^b^	0.97 ± 0.01 ^a^	0.67 ± 0.01 ^c^	0.36 ± 0.01 ^d^
	Glucosidase inhibition (mmol ACAE/g)	0.0001	0.91 ± 0.02 ^b^	na	0.97 ± 0.01 ^a^	na
	Elastase inhibition (mmol CE/g)	0.0001	1.07 ± 0.07 ^b^	1.98 ± 0.10 ^a^	2.21 ± 0.12 ^a^	2.08 ± 0.06 ^a^

* Values are reported as mean ± S.D. TE: Trolox equivalent; EDTAE: EDTA equivalent; GALAE: Galatamine equivalent; KAE: Kojic acid equivalent; ACAE: Acarbose equivalent. na: not active. a–d. line wise values with same superscripts of this type indicate no significant difference among extracts (*p* > 0.05).

**Table 5:** Antifungal activity of *P. kotschyi* samples (mg/mL) *.

	Samples	*A.v.*	*A.o.*	*A.n.*	*A.f.*	*P.o.*	*P.f.*	*T.v.*	*P.v.c*
Minimum inhibitory concentration (MIC)	Leaves Methanol	0.21 ± 0.01 ^a^	0.10 ± 0.01 ^c^	0.15 ± 0.01 ^b^	0.20 ± 0.01 ^a^	0.20 ± 0.01 ^b^	0.15 ± 0.01 ^b^	0.10 ± 0.01 ^c^	0.30 ± 0.01 ^a^
	Leaves Water	0.10 ± 0.01 ^c^	0.10 ± 0.01 ^c^	0.15 ± 0.01 ^b^	0.21 ± 0.01 ^a^	0.10 ± 0.01 ^d^	0.10 ± 0.01 ^c^	0.11 ± 0.01 ^c^	0.20 ± 0.01 ^b^
	Stem Bark Methanol	0.15 ± 0.01 ^b^	0.15 ± 0.01 ^b^	0.20 ± 0.01 ^a^	0.15 ± 0.01 ^b^	0.20 ± 0.01 ^b^	0.20 ± 0.01 ^a^	0.15 ± 0.01 ^b^	0.20 ± 0.01 ^b^
	Stem Bark Water	0.15 ± 0.01 ^b^	0.15 ± 0.01 ^b^	0.21 ± 0.01 ^a^	0.11 ± 0.01 ^c^	0.15 ± 0.01 ^c^	0.15 ± 0.01 ^b^	0.11 ± 0.01 ^c^	0.20 ± 0.01 ^b^
	Ketoconazole	0.20 ± 0.01 ^a^	0.20 ± 0.01 ^a^	0.15 ± 0.01 ^b^	0.20 ± 0.01 ^a^	1.00 ± 0.01 ^a^	0.20 ± 0.01 ^a^	1.00 ± 0.01 ^a^	0.20 ± 0.01 ^b^
	Bifonazole	0.10 ± 0.01 ^c^	0.15 ± 0.01 ^b^	0.10 ± 0.01 ^c^	0.15 ± 0.01 ^b^	0.20 ± 0.01 ^b^	0.20 ± 0.01 ^a^	0.15 ± 0.01 ^b^	0.10 ± 0.01 ^c^
Minimum fungicidal concentration (MFC)	Leaves Methanol	0.40 ± 0.01 ^b^	0.40 ± 0.01 ^b^	0.21 ± 0.01 ^b^	0.41 ± 0.01 ^b^	0.41 ± 0.01 ^b^	0.20 ± 0.01 ^d^	0.20 ± 0.01 ^c^	0.40 ± 0.01 ^a^
	Leaves Water	0.21 ± 0.01 ^d^	0.20 ± 0.01 ^d^	0.20 ± 0.01 ^b^	0.41 ± 0.01 ^b^	0.21 ± 0.01 ^e^	0.21 ± 0.01 ^d^	0.20 ± 0.01 ^c^	0.41 ± 0.01 ^a^
	Stem Bark Methanol	0.30 ± 0.01 ^c^	0.30 ± 0.01 ^c^	0.31 ± 0.01 ^a^	0.30 ± 0.01 ^c^	0.30 ± 0.01 ^c^	0.30 ± 0.01 ^b^	0.30 ± 0.01 ^b^	0.30 ± 0.01 ^b^
	Stem Bark Water	0.30 ± 0.01 ^c^	0.31 ± 0.01 ^c^	0.30 ± 0.01 ^a^	0.15 ± 0.01 ^e^	0.30 ± 0.01 ^c^	0.31 ± 0.01 ^b^	0.15 ± 0.01 ^d^	0.30 ± 0.01 ^b^
	Ketoconazole	0.50 ± 0.01 ^a^	0.50 ± 0.01 ^a^	0.20 ± 0.01 ^b^	0.50 ± 0.01 ^a^	1.50 ± 0.01 ^a^	0.50 ± 0.01 ^a^	1.50 ± 0.01 ^a^	0.30 ± 0.01 ^b^
	Bifonazole	0.20 ± 0.01 ^d^	0.20 ± 0.01 ^d^	0.15 ± 0.01 ^c^	0.20 ± 0.01 ^d^	0.25 ± 0.01 ^d^	0.25 ± 0.01 ^c^	0.20 ± 0.01 ^c^	0.20 ± 0.01 ^c^

* Different letters indicate the differences in these samples for each fungal strains (*p* < 0.05). Statistical analysis were individually performed for minimum inhibitory concentration (MIC) and minimum fungicidal concentration (MFC) values. *A.v.*: *Aspergillus versicolor*; *A.o.*: *Aspergillus ochraceus*; *A.n.*: *Aspergillus niger*; *A.f.*: *Aspregillus fumigatus*; *P.o.*: *Penicillium ochrochloron*; *P.f.*: *Penicillium funiculosum*; *T.v*: *Trichoderma viride*; *P.v.c.*: *Penicillium verrucosum var. cyclopium*.

**Table 6:** Antibacterial activity of *P. kotschyi* samples (mg/mL) *.

	Samples	*B.c.*	*M.f.*	*S.a.*	*L.m.*	*E. coli*	*P.a.*	*E.c.*	*S.t.*
Minimum inhibitory concentration (MIC)	Leaves Methanol	0.15 ± 0.01 ^b^	0.31 ± 0.01 ^a^	0.10 ± 0.01 ^b^	0.05 ± 0.01 ^c^	0.30 ± 0.01 ^b^	0.30 ± 0.01 ^a^	0.61 ± 0.01 ^a^	0.60 ± 0.01 ^a^
	Leaves Water	0.15 ± 0.01 ^b^	0.30 ± 0.01 ^a^	0.21 ± 0.01 ^a^	0.20 ± 0.01 ^a^	0.31 ± 0.01 ^b^	0.30 ± 0.01 ^a^	0.20 ± 0.01 ^b^	0.15 ± 0.01 ^b^
	Stem Bark Methanol	0.11 ± 0.01 ^c^	0.04 ± 0.01 ^d^	0.04 ± 0.01 ^c^	0.04 ± 0.01 ^d^	0.11 ± 0.01 ^d^	0.11 ± 0.01 ^c^	0.03 ± 0.01 ^e^	0.07 ± 0.01 ^d^
	Stem Bark Water	0.20 ± 0.01 ^a^	0.31 ± 0.01 ^a^	0.03 ± 0.01 ^c^	0.21 ± 0.01 ^a^	0.40 ± 0.01 ^a^	0.15 ± 0.01 ^b^	0.15 ± 0.01 ^c^	0.16 ± 0.01 ^b^
	Streptomycin	0.10 ± 0.01 ^d^	0.05 ± 0.01 ^c^	0.10 ± 0.01 ^b^	0.15 ± 0.01 ^b^	0.10 ± 0.01 ^d^	0.10 ± 0.01 ^d^	0.10 ± 0.01 ^d^	0.10 ± 0.01 ^c^
	Ampicillin	0.10 ± 0.01 ^d^	0.10 ± 0.01 ^b^	0.10 ± 0.01 ^b^	0.15 ± 0.01 ^b^	0.15 ± 0.01 ^c^	0.30 ± 0.01 ^a^	0.15 ± 0.01 ^c^	0.10 ± 0.01 ^c^
Minimum bactericidal concentration (MBC)	Leaves Methanol	0.20 ± 0.01 ^b^	0.40 ± 0.01 ^b^	0.20 ± 0.01 ^b^	0.21 ± 0.01 ^d^	0.40 ± 0.01 ^b^	0.40 ± 0.01 ^b^	0.80 ± 0.01 ^a^	0.80 ± 0.01 ^a^
	Leaves Water	0.20 ± 0.01 ^b^	0.41 ± 0.01 ^b^	0.40 ± 0.01 ^a^	0.40 ± 0.01 ^b^	0.41 ± 0.01 ^b^	0.41 ± 0.01 ^b^	0.40 ± 0.01 ^c^	0.20 ± 0.01 ^c^
	Stem Bark Methanol	0.15 ± 0.01 ^c^	0.07 ± 0.01 ^e^	0.08 ± 0.01 ^d^	0.08 ± 0.01 ^e^	0.15 ± 0.01 ^d^	0.16 ± 0.01 ^e^	0.07 ± 0.01 ^e^	0.15 ± 0.01 ^d^
	Stem Bark Water	0.30 ± 0.01 ^a^	0.60 ± 0.01 ^a^	0.07 ± 0.01 ^d^	0.30 ± 0.01 ^c^	0.61 ± 0.01 ^a^	0.31 ± 0.01 ^c^	0.60 ± 0.01 ^b^	0.30 ± 0.01 ^b^
	Streptomycin	0.15 ± 0.01 ^c^	0.10 ± 0.01 ^d^	0.20 ± 0.01 ^b^	0.30 ± 0.01 ^c^	0.20 ± 0.01 ^c^	0.20 ± 0.01 ^d^	0.20 ± 0.01 ^d^	0.20 ± 0.01 ^c^
	Ampicillin	0.15 ± 0.01 ^c^	0.15 ± 0.01 ^c^	0.15 ± 0.01 ^c^	0.50 ± 0.01 ^a^	0.20 ± 0.01 ^c^	0.50 ± 0.01 ^a^	0.20 ± 0.01 ^d^	0.20 ± 0.01 ^c^

* Column wise values with different letters (a–e) indicate the differences in these samples for each bacterial strains (*p* < 0.05). Statistical analysis were individually performed for minimum inhibitory concentration (MIC) and minimum bactericidal concentration (MBC) values. *B.c.*: *Bacillus cereus*; *M.f.*: *Micrococcus flavus*; *S.a.*: *Staphylococcus aureus*; *L.m.*: *Listeria monocytogenes*; *E. coli*: *Escherichia coli*; *P.a.*: *Pseudomonas aeruginosa*; *E.c*: *Enterobacter cloacae*; *S.t*.: *Salmonella typhimurium*.

## Data Availability

Data is contained within the article.
